# ZIF-67 Anchored on MoS_2_/rGO Heterostructure for Non-Enzymatic and Visible-Light-Sensitive Photoelectrochemical Biosensing

**DOI:** 10.3390/bios14010038

**Published:** 2024-01-12

**Authors:** Qiaolin Fan, Xiao Li, Hui Dong, Zhonghua Ni, Tao Hu

**Affiliations:** School of Mechanical Engineering, Jiangsu Key Laboratory for Design and Manufacture of Micro-Nano Biomedical Instruments, Southeast University, Nanjing 211189, China; 220210276@seu.edu.cn (Q.F.); 220190306@seu.edu.cn (H.D.); nzh2003@seu.edu.cn (Z.N.)

**Keywords:** ZIF-67, 2D material, non-enzymatic, heterostructure, visible-light sensitive, photoelectrochemical biosensing

## Abstract

Graphene and graphene-like two-dimensional layered nanomaterials-based photoelectrochemical (PEC) biosensors have recently grown rapidly in popularity thanks to their advantages of high sensitivity and low background signal, which have attracted tremendous attention in ultrahigh sensitive small molecule detection. This work proposes a non-enzymatic and visible-light-sensitive PEC biosensing platform based on ZIF-67@MoS_2_/rGO composite which is synthesized through a facile and one-step microwave-assisted hydrothermal method. The combination of MoS_2_ and rGO could construct van der Waals heterostructures, which not only act as visible-light-active nanomaterials, but facilitate charge carriers transfer between the photoelectrode and glassy carbon electrode (GCE). ZIF-67 anchored on MoS_2_/rGO heterostructures provides large specific surface areas and a high proportion of catalytic sites, which cooperate with MoS_2_ nanosheets, realizing rapid and efficient enzyme-free electrocatalytic oxidation of glucose. The ZIF-67@MoS_2_/rGO-modified GCE can realize the rapid and sensitive detection of glucose at low detection voltage, which exhibits a high sensitivity of 12.62 μAmM^−1^cm^−2^. Finally, the ZIF-67@MoS_2_/rGO PEC biosensor is developed by integrating the ZIF-67@MoS_2_/rGO with a screen-printed electrode (SPE), which exhibits a high sensitivity of 3.479 μAmM^−1^cm^−2^ and a low detection limit of 1.39 μM. The biosensor’s selectivity, stability, and repeatability are systematically investigated, and its practicability is evaluated by detecting clinical serum samples.

## 1. Introduction

Glucose is an important biomarker in the clinical detection of hyperglycemia and diabetes. Up to now, there have been many methods for glucose detection, including fluorescence detection [1], Raman spectroscopy detection [2] and colorimetric methods [3], electrochemical detection [4,5,6], photoelectrochemical (PEC) detection [7], etc., by analyzing the correlation between different signals and glucose concentration. Among them, the PEC technique has received tremendous research interest due to its merits of fast response, low background signal, and high sensitivity, which have been extensively investigated in the fields of protein, nucleic acid, biomolecules, and metal ions [8,9,10,11]. Compared with the electrochemical method, it should work with a light source which is utilized to excite the electrode to produce photogenerated electron–hole pairs, and then the electrons are transferred to the surface of the electrode, achieving the conversion of photo energy to electrical energy.

The photogenerated charge carriers are critical to the photocurrent in the PEC detection process; hence, developing photoactive materials and constructing highly sensitive photoelectrode are significant in PEC research. Until now, semiconductors such as ZnO and TiO_2_ have been thoroughly studied due to their high activity, low cost, and stable chemical and physical properties [12,13]. While limited by their wide band gaps, they present poor absorption of visible light and rapid combination of photogenerated carriers. To solve these problems, many efforts have been made to broaden light absorption to the visible spectral region. Two-dimensional layered nanomaterials, such as graphene, reduced graphene oxide (rGO), and transition metal dichalcogenides (TMDs), are suitable candidates to build van der Waals heterostructures through a “bottom-up” strategy with high specific surface area, fast electron transfer rate, and good light harvest property [14]. Among them, MoS_2_ is one of the most typical TMDs with a tunable band gap greatly correlative to layer numbers (about 1.2~1.9 eV). Compared to semiconductor materials with a wide band gap, MoS_2_ can increase the absorption of visible light and promote charge transfer rates [15]. In addition, graphene or rGO earns a widespread reputation due to its excellent electron mobility and high specific surface area, which are suitable to conquer the high recombination of photogenerated electron –hole pairs. It is a promising approach to combine MoS_2_ with graphene or rGO and form a layered heterostructure, which will largely improve the absorption of visible light and accelerate the separation of electron–hole pairs at the same time.

Another efficient strategy to improve the sensitivity of PEC biosensors is to introduce promoters of signal amplification. Using enzyme-catalyzed substrate is an effective way to realize signal amplification by introducing enzymes on photoelectrodes to catalyze analytes and increase detection current. Glucose oxidase is highly selective and sensitive, can detect glucose quickly and accurately, and plays a key role in the production of many glucose sensors [16,17,18,19]. However, enzymes are susceptible to denaturation and are affected by temperature, pH, and environmental conditions, which is far from satisfactory to realize biosensing [20,21,22]. At present, many efforts have been made to explore enzyme-free and sensitive electrode materials. Notably, metal-organic frameworks (MOFs) are a class of crystalline porous materials with a periodic network structure formed by the self-assembly of inorganic metal centers and organic ligands; they demonstrate high electrocatalytic activity and selectivity toward glucose [23,24]. Among a series of MOFs, zeolitic imidazolate frameworks (ZIFs), which are topologically isomorphic with zeolites, inherit all the advantages of MOFs in the areas of electrocatalysis and biosensing [25]. Both MOFs and ZIFs are mainly prepared by conventional hydrothermal or solvothermal methods, which have deficiencies such as a long preparation time and complicated process. Therefore, it is necessary to develop an efficient material synthesis method to prepare enzyme-free electrode materials with a high performance in glucose detection. Microwave-assisted synthesis utilizes microwaves and the coupling of reaction media on materials to produce high-energy electromagnetic radiation and effective internal heating [26]. Compared with the traditional hydrothermal method, it demonstrates multiple benefits, including a higher heating efficiency, lower cost of synthetic material, more simple operation of a microwave synthesizer, and effective control of the reaction parameters, which can control the process of crystallization and obtain the desired structure and morphology [27,28].

Based on the above considerations, this work adopted a microwave-assisted synthesis method to synthesize a ZIF-67@MoS_2_/rGO enzyme-free photoelectrode in one step as well as and develop a visible-light-sensitive lab-on-chip PEC biosensing platform. Firstly, ZIF-67@MoS_2_/rGO was modified on GCE, and its high performance for glucose detection including a high sensitivity, wide detection range, and low detection limit were verified by PEC methods. The rGO serves as a carrier transport layer, and the heterostructure formed between rGO and MoS_2_ presents a photoelectrical transducer that generates and transports photocarriers. The synergy electrocatalytic effect between MoS_2_ and ZIF-67 proved to be crucial for glucose sensing and signal amplification. Then, a ZIF-67@MoS_2_/rGO PEC glucose biosensor was developed by integrating the photoactive materials with SPE, suggesting good potential for biochemical applications.

## 2. Materials and Methods

### 2.1. Reagents and Chemicals

Graphene oxide was purchased from Nanjing XFNANO (Nanjing, China). 2-methylimidazole, lactose, maltose, and fructose were purchased from Aladdin (Shanghai, China). Co(NO_3_)_2_•6H_2_O, L-Cysteine, Thiourea, NaOH, and NaCl were bought from HUSHI (Shanghai, China). Glucose was obtained from Shanghai Sigma Aldrich trading Co., Ltd. (Shanghai, China). All other reagents of guaranteed reagent (GR) level were obtained from Sigma. Double distilled water (ultra-pure conductivity of ≥18 MΩ) was used for all experimental analysis.

### 2.2. Instrumentation and Measurements

Scanning electron microscopy (SEM) was carried out on a FEI Quanta 200 microscope (FEI company, Hillsboro, USA). X-ray diffraction (XRD) and Raman spectra were acquired using Ultima IV (Rigaku Corporation, Tokyo, Japan) and WITec Alpha300R (WITec GmbH, Ulm, Germany), respectively. CV experiments were performed with a CHI 660D electrochemical workstation (Shanghai CH Instrument Company, Shanghai, China). A three-electrode configuration, using Ag/AgCl (3 M KCl saturated) as the reference electrode, platinum sheet as the counter electrode, and photoactive material-modified GCE as the working electrode, was adopted for electrochemical tests in a freshly prepared sodium hydroxide electrolyte solution (NaOH, 0.1 M). The light source was provided by xenon lamp illumination CEL-HXF300 (Beijing China Education Au-light Co., Ltd., Beijing, China). Optical filters UVIRCUT 420 (Beijing China Education Au-light Co., Ltd., Beijing, China) were used to obtain a visible-light of wavelength between 420 and 780 nm.

### 2.3. Synthesis of ZIF-67@MoS_2_/rGO Composite

First, 0.8 g thiourea and 0.4 g ammonium molybdate were measured and slowly poured into 10 mL of deionized water to make solution A. Next, 1.35 mM Co(NO_3_)_2_•6H_2_O and 8.1 mM 2-methylimidazole were dissolved in 4.5 mL of methyl alcohol evenly to form solution B. Then, 5 mL of graphene oxide was added to solution A, and solution B was slowly poured into solution A. The mixture was stirred at 900 rpm for 30 min and ultrasonized for 1 h, then it underwent microwave-assisted heating at 180 °C for 1 h. The product was centrifugated at 12,000 rpm to obtain precipitate, washed with methanol and ethanol alternately, and finally dried at 60 °C thoroughly to obtain ZIF-67@MoS_2_/rGO powder. In addition, MoS_2_ and MoS_2_/rGO were synthesized through the same procedures for comparison.

### 2.4. Preparation of ZIF-67@MoS_2_/rGO-Modified Photoelectrode

The obtained ZIF-67@MoS_2_/rGO powder was dissolved in ethanol solution, and 5 μL Nafion was added. The solution was ultrasonicated to dissolve evenly. The glassy carbon electrode (GCE) with a diameter of 4 mm was sanded and washed, then the as-prepared ZIF-67@MoS_2_/rGO-Nafion solution was dropped onto the electrode and dried at room temperature to make the ZIF-67@MoS_2_/rGO-modified glassy carbon electrode (ZIF-67@MoS_2_/rGO GCE). ZIF-67@MoS_2_/rGO powder was also dissolved in deionized water and modified on the screen-printed electrode (SPE) following the same procedure to make the ZIF-67@MoS_2_/rGO-modified screen-printed electrode (ZIF-67@MoS_2_/rGO SPE). 

## 3. Results and Discussion

### 3.1. Materials Characterization

**Figure 1:**
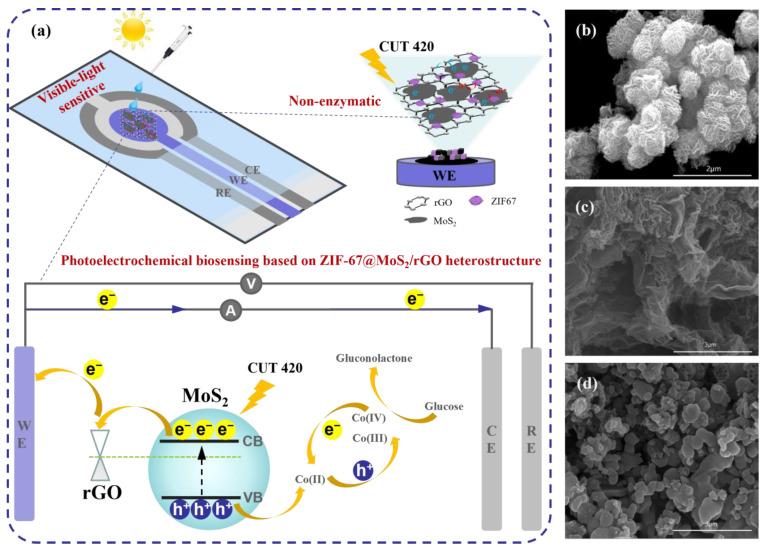
(**a**) Schematic diagram of the visible-light-sensitive PEC biosensing based on non-enzymatic ZIF-67@MoS_2_/rGO; SEM characterization of (**b**) MoS_2_, (**c**) MoS_2_/rGO, and (**d**) ZIF-67@MoS_2_/rGO.

**Figure 2:**
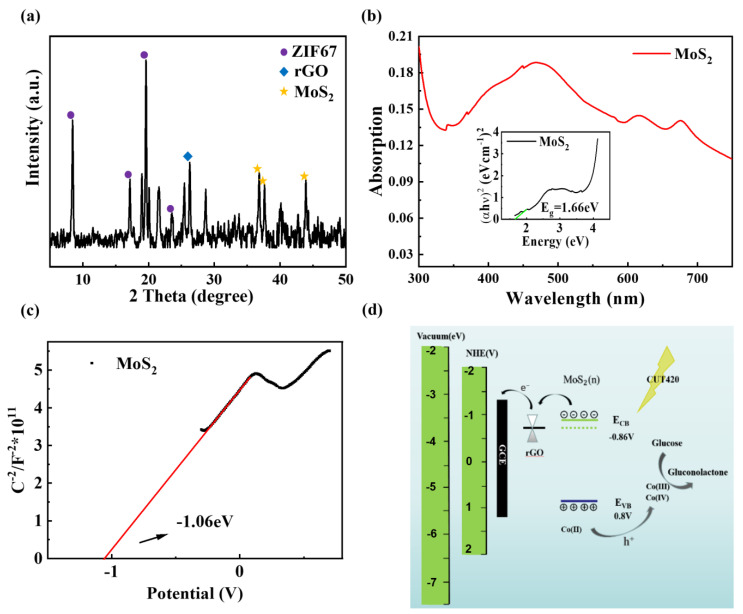
(**a**) XRD of ZIF-67@MoS_2_/rGO. (**b**) UV-visible absorption spectrum and Tauc diagram of MoS_2_; (**c**) M-S diagram of MoS_2_. (**d**) PEC biosensing mechanism diagram of ZIF-67@MoS_2_/rGO.

Figure 1a demonstrates a schematic diagram of the visible-light-sensitive PEC biosensing based on non-enzymatic ZIF-67@MoS_2_/rGO. In order to verify the rationality of this microwave-assisted hydrothermal synthesis method, SEM, XRD, and Raman spectroscopy were used to characterize MoS_2_, ZIF-67/MoS_2_, and ZIF-67@MoS_2_/rGO. SEM characterizations of MoS_2_, MoS_2_/rGO, and ZIF-67@MoS_2_/rGO are shown in Figure 1b–d. MoS_2_ synthesized by this method had a flower cluster-shaped structure, providing a large specific surface area for better light absorption. The introduction of rGO made the flower clusters transform to dense lamellas spreading over a layered microstructure, leading to a more extended structure of MoS_2_/rGO, which created rich pathways for charge transport. ZIF-67@MoS_2_/rGO demonstrated a distinct hierarchical geometric structure, which is a typical structure of ZIF-67. This morphology indicated that ZIF-67 totally covers MoS_2_/rGO hybrid nanosheets, and the change in morphology characteristics proved that the ZIF-67@MoS_2_/rGO composite structure was successfully synthesized. As shown in Figure 2a, the synthesis of ZIF-67@MoS_2_/rGO was further analyzed by X-ray diffraction (XRD). Furthermore, 36.8°, 39.98°, and 44.3° correspond to the peak value of MoS_2_, while 26.28° is the characteristic peak of rGO [29,30]. In addition to these peaks, the prominent peaks at 7.66°, 17.1°, 19.72°, and 25.42° in the composite material correspond to ZIF-67 [31].

### 3.2. Photoelectrochemical Property Characterization

Chronoamperometry was used to investigate the photocurrent of the GCEs modified with different photosensitive materials. We systematically optimized the experimental conditions, including the detection potential, optical power density, and pH of electrolytes (See Figure S2 for details). The detection potential, light source intensity, and pH value affect the recombination of photogenerated electrons and holes in photoelectrochemistry. Therefore, as shown in Figure S2a, the photocurrent response of ZIF-67@MoS_2_/rGO GCE to 0.5 mM glucose was comprehensively compared in a potential range of 0.1–0.5 V. At 0.1–0.3 V, and the photocurrent response of the electrode was greatly improved with the increase in detection potential, which was because the increased detection potential promoted the separation of photogenerated electrons and holes and improved the response of the electrode [32]. Above 0.3 V, the increase in the electrode photocurrent response slows down. We considered the trade-off effect between high photocurrent response and negative influences brought by high potential, and 0.3 V was finally selected as the detection potential for subsequent tests.

Light source intensity is very important to photocurrent response as the excitation device of photochemistry. In this paper, the optical power density of xenon lamp source was optimized, and different test light intensities of 70–100 mWcm^−2^ were selected to compare the photocurrent response of the electrode, as shown in Figure S2b. With the increase in optical power density, the photocurrent response increased and gradually reached light saturation. However, when the optical power density increased further, the sensitivity of electrode detection could not be improved. As there was no sacrificial agent added to the system, excessive light would produce a large number of holes, which could rapidly corrode the material or cause sample evaporation [33]. Therefore, 100 mWcm^−2^ was finally selected as the optimal light intensity for testing.

We also conducted PEC detection on electrolytes with different pH values, and the photocurrent responses with the pH values changing from 10 to 14 at 0.3 V were recorded in Figure S2c. As pH value continued to rise, OH^−^ promoted the catalytic oxidation of glucose, increasing the photocurrent correspondingly. However, when the pH value increased to 1 M, the photocurrent signal showed a downward trend, which may be because the high NaOH concentration under light intensified the disproportionation reaction of glucose and reduced the glucose concentration in the system [34], resulting in a downward trend in photocurrent. Therefore, the electrolyte with a 0.1 M NaOH concentration was selected as the optimal test environment. In the subsequent investigation, 0.3 V was selected as the optimal applied potential, and 0.1 M NaOH was selected as the optimal test environment with 100 mWcm^−2^ light intensity.

In 0.1 M NaOH under visible-light on/off illumination, ZIF-67 GCE, ZIF-67/MoS_2_ GCE, and ZIF-67@MoS_2_/rGO GCE were tested containing 0.1 mM glucose and 0.2 mM glucose, respectively (Figure S3). As shown in Figure S3a, ZIF-67 GCE demonstrates a slight rise in photocurrent, which was due to a small amount of catalytic reaction between ZIF-67 and glucose in the alkaline environment. However, the photocurrent shows a downward trend immediately, and when the glucose concentration changes from 0.1 mM to 0.2 mM, current increment even declined to a small value. When ZIF-67 is combined with MoS_2_ (Figure S3b), as MoS_2_ is a narrow-band gap semiconductor with an appropriate band gap, it can better absorb light and generate photogenerated electron–hole pairs. At the same time, MoS_2_ has a certain number of catalytic sites, and so the current increments are more obvious and show a certain correlation with glucose concentration; meanwhile, due to the inferior conductivity of MoS_2_, photogenerated electron–hole pairs generated by MoS_2_ are more likely to recombined. When introducing rGO nanosheets, their high conductivity will promote electron transfer to GCEs quickly, largely restricting the combination of photogenerated charges, so the photocurrent responses of ZIF-67@MoS_2_/rGO GCE in Figure S3c are significantly improved. Finally, current increments at the potential of 0.3 V for ZIF-67-, ZIF-67/MoS_2_-, and ZIF-67@MoS_2_/rGO-modified GCEs in 0.1 mM and 0.2 mM glucose are summarized in Figure S3d, and are consistent with the above analysis. As shown in Figure S3, the photocurrent response of ZIF-67@MoS_2_/rGO GCE is more significant, indicating that it is crucial to decorate ZIF-67 on MoS_2_/rGO heterostructures for glucose detection.

To uncover the PEC biosensing mechanism of ZIF-67@MoS_2_/rGO, first the UV-visible absorption spectrum of MoS_2_ was measured by UV spectrophotometer, and multiple absorption peaks located at the visible-light range were identified, indicating MoS_2_ acts as the photoactive component (Figure 2b). The Tauc diagram of MoS_2_ was converted by its UV-visible absorption spectrum; the X-axis was Energy (hυ), which was 1240/wavelength; and the Y-axis was calculated on the basis of (αhυ)^n^ = k(hυ − E_g_) [35]. In this formula, n is 2, α is 2.303, and the band gap E_g_ of the material is finally obtained as 1.66 eV. The M-S diagram of MoS_2_ is shown in Figure 2c, and the value at the intersection between the M-S diagram and the X-axis is the flat band potential, which was calculated according to the conversion formula: E(NHE) = E(Ag/AgCl) + 0.2. In addition, the type of semiconductor was determined by the slope [36]. Hence, the results show that, in this work, MoS_2_ is an N-type semiconductor, with its Fermi energy level close to the conduction band and its conduction band at position E_CB_ = −0.86 eV, together with its valence band at position E_VB_ = 0.8 eV.

Figure 2d shows the reaction mechanism of the ZIF-67@MoS_2_/rGO photoelectrode. Through MoS_2_’s sufficient absorption of visible light, the photogenerated holes promote the oxidation of Co(II) and Co(III) to Co(III) and Co(IV) in ZIF-67, respectively. The processes can be expressed by the following formula:

Co(II)-ZIF + h+ → Co(III)-ZIF(1)

Co(III)-ZIF + h+ → Co(IV)-ZIF(2)

Subsequently, glucose is oxidized by Co(III) and Co(IV), and then hypervalent cobalt compounds are reduced to their original states. The catalytic mechanism of glucose under light conditions can be presumably proposed as the following equations:

Co(III)-ZIF + C_6_H_12_O_6_ → Co(II)-ZIF + C_6_H_10_O_6_ + H_2_O (3)

Co(IV)-ZIF + C_6_H_12_O_6_ → Co(III)-ZIF + C_6_H_10_O_6_ + H_2_O (4)

**Figure 3:**
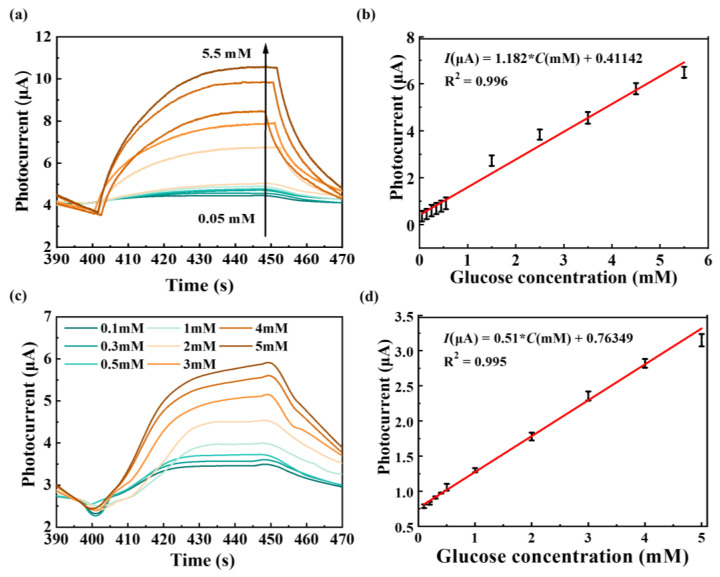
(**a**) The photocurrent–time curves of ZIF-67@MoS_2_/rGO GCEs with the successive injections of glucose into 0.1 M NaOH electrolyte. (**b**) The linear fitting of photocurrent vs. glucose concentration corresponding to (**a**). (**c**) The photocurrent–time curves of ZIF-67@MoS_2_/rGO SPEs with the successive injection of glucose into 0.1 M NaOH electrolyte. (**d**) The linear fitting of photocurrent vs. glucose concentration corresponding to (**c**).

Photocurrent responses of ZIF-67@MoS_2_/rGO in 0.1 M NaOH containing 0.05–0.55 mM glucose (low concentration range, 0.1 mM as an interval) and 1.5–5.5 mM glucose (high concentration range, 1 mM as an interval) are plotted in Figure 3a. With the increase in glucose concentration, the photocurrent responses gradually increase and exhibit a good correlation with glucose concentration. The obtained photocurrents were fitted with the corresponding glucose concentration, and the result is shown in Figure 3b. The photocurrent responses of ZIF-67@MoS_2_/rGO have a good linear relationship in the concentration range of 0.05–4.5 mM, and their sensitivity is 12.62 μAmM^−1^cm^−2^. The limit of detection (LOD) was calculated as 3.9 μM (S/N = 3) according to the slope in the calibration curve of glucose detection and instrument noise. The repeatability and selectivity of ZIF-67@MoS_2_/rGO GCE are shown in Figures S4 and S5. Table S1 shows the comparison of ZIF-67@MoS_2_/rGO GCE and other photoelectrochemical glucose sensors [37,38,39,40,41,42], and the practical application of ZIF-67@MoS_2_/ rGO GCEs are investigated by testing glucose concentrations in human serum samples (Table S2).

In this work, a glucose biosensor based on a screen-printed electrode (SPE) was further developed. The as-synthesized ZIF-67@MoS_2_/rGO powder was dissolved in deionized water to make a 3 mg/mL aqueous solution of ZIF-67@MoS_2_/rGO, and 5 μL Nafion solution was added to the solution, which was fully dissolved in ultrasound. Then, 10 μL ZIF-67@MoS_2_/rGO was dropped on the working electrode and dried to obtain ZIF-67@MoS_2_/rGO SPE. As before, in 0.1 M NaOH under visible-light on/off illumination, the response photocurrent obtained at glucose concentrations of 0.1 mM, 0.3 mM, 0.5 mM, 1 mM, 2 mM, 3 mM, 4 mM, and 5 mM with an applied potential of 0.3 V were are in Figure 3c. The photocurrent responses to SPE also increased with the increase in glucose concentration. The obtained photocurrents were fitted with the corresponding glucose concentration, and the results are shown in Figure 3d. The ZIF-67@MoS_2_/rGO SPE has better glucose detection characteristics, and the photocurrent responses have a good linear relationship with glucose concentration ranging from 0.1 mM to 5 mM. The sensitivity was 3.479 μAmM^−1^cm^−2^ and the LOD was calculated as 1.39 μM (S/N = 3), according to the slope in the calibration curve of glucose detection and instrument noise. A comparison of the ZIF-67@MoS_2_/rGO SPE glucose sensor and other lab-on-chip PEC glucose sensors is shown in Table S3 [43,44,45,46,47], indicating that our strategy is competitive and feasible for PEC glucose sensing.

### 3.3. Interference Studies and Practical Applications

**Figure 4:**
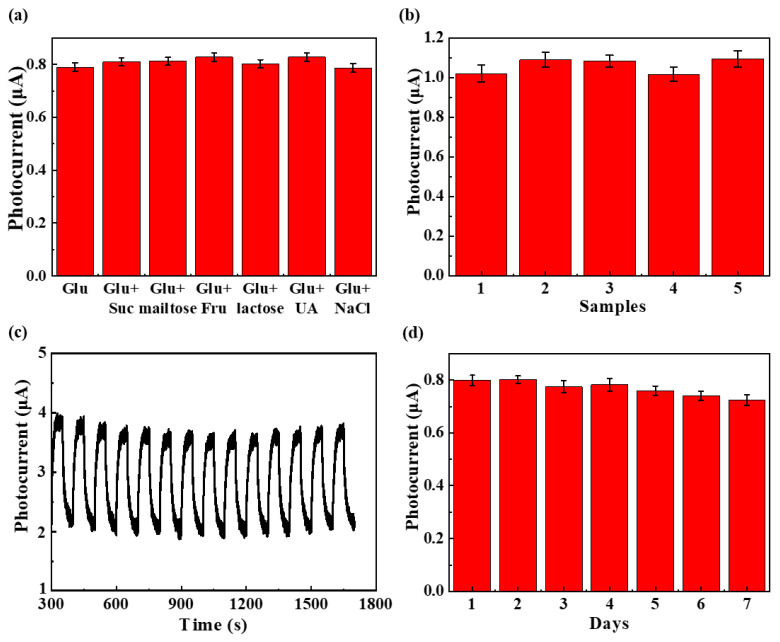
(**a**) Amperometric response of ZIF-67@MoS_2_/rGO SPE to 0.1 mM glucose with other interfering substances such as sucrose, maltose, fructose, lactose, uric acid, and NaCl in 0.1 M NaOH. (**b**) The repeatability of ZIF-67@MoS_2_/rGO SPEs to 0.5 mM glucose. Photocurrent responses of ZIF-67@MoS_2_/rGO SPE for (**c**) cyclic test of 1400 s. (**d**) Test for 7 consecutive days.

The selectivity of ZIF-67@MoS_2_/rGO SPE for glucose detection was evaluated by an interference test. In this experiment, 0.1 mM glucose with 0.5 mM sucrose, maltose, fructose, lactose, uric acid, and NaCl were added to 0.1 M NaOH solution, respectively. After stabilization, the photocurrent responses were recorded, as shown in Figure 4a. The results show that this photoelectrode has good selectivity for glucose detection. To further evaluate its repeatability, five different ZIF-67@MoS_2_/rGO SPEs were tested against 0.5 mM glucose in 0.1 M NaOH electrolyte, and the photocurrent responses at 0.3 V were recorded for comparison (Figure 4b). The relative standard deviations (RSD) of the photocurrent in response to 0.5 mM glucose were 1.5%, respectively, showing the acceptable repeatability of ZIF-67@MoS_2_/rGO SPEs.

The stability of ZIF-67@MoS_2_/rGO SPE in 0.1 M NaOH under long-term illumination was investigated. As shown in Figure 4c, after the cyclic test of 1400 s, the photocurrent response was still 98.6% of its initial value, which proves that ZIF-67@MoS_2_/rGO has good stability under long-term strong-light irradiation. Meanwhile, the photoelectrode was tested for 7 consecutive days, and the results are recorded in Figure 4d. The photocurrent response was still 94.3% of its initial value. Therefore, it can be concluded that ZIF-67@MoS_2_/rGO SPE can still maintain good performance after a certain period of daily storage, even if there is some occasional interference of ambient light.

The practical application of ZIF-67@MoS_2_/rGO SPE was investigated by testing glucose concentrations in human serum samples, which were provided by Zhongda Hospital in Nanjing, China. Before PEC testing, the cryo-preserved sample was thawed at room temperature, and a certain amount was added into 0.1 M NaOH with a pipetting gun to form 5% human serum sample. The spike and recovery method are employed, and the analytical results are listed in Table 1. The obtained results suggest that the developed PEC biosensor based on ZIF-67@MoS_2_/rGO SPE exhibits high suitability for glucose detection in genuine samples, offering high potential for practical applications in clinical diagnosis.

Compared with the electrochemical detection method, the PEC detection of glucose has lower background signal and a higher sensitivity due to its different excitation signal and detection signal. It further improves the convenience and instantaneity of detection when combined with the screen-printed electrode, which is simple to produce in bulk, and has a wide range of application prospects.

## 4. Conclusions

In this work, ZIF-67@MoS_2_/rGO was synthesized by a facile and one-step microwave-assisted hydrothermal method for the PEC detection of glucose. Due to the excellent visible-light absorption of MoS_2_ and the enhancement of photogenerated electron transfer characteristics of rGO, MoS_2_/rGO hybrid nanosheets constructed a suitable heterostructure for PEC biosensing. ZIF-67 anchored on MoS_2_/rGO composite could realize the efficient, enzyme-free, electrocatalytic oxidation of glucose, achieving a high sensitivity of 12.62 μAmM^−1^cm^−2^ based on GCEs. To further improve the portability and immediacy of glucose detection, ZIF67@MoS_2_/rGO was integrated with SPE to develop a lab-on-chip PEC biosensor. The as-prepared biosensor exhibited a competitive sensitivity of 3.479 μAmM^−1^cm^−2^ and low detection limit of 1.39 μM, which also demonstrates its outstanding selectivity, stability, and repeatability. Finally, clinical serum samples were tested to verify its practicability, and the obtained results suggest that this PEC biosensor has a good development prospect for clinical applications in quantitative detection of biomolecules.

## Figures and Tables

**Table 1:** PEC glucose detection in 5% human serum with ZIF-67@MoS_2_/rGO-modified SPE.

Real Sample	Fitted Value (μA)	Scalar Addition (μA)	Estimated Value (μA)	Recovery (%)
1	0.784	1.3	2.135	103.9
2	0.833	1.3	2.166	102.5
3	0.792	1.3	2.045	96.4

## Data Availability

The data presented in this study are available on request from the corresponding author.

## Supplementary Materials

The following supporting information can be downloaded at: https://www.mdpi.com/article/10.3390/bios14010038/s1, Figure S1: Schematic diagram of the photoelectrochemical laboratory bench; Figure S2: Optimization of (a) detection potential; (b) optical power density; (c) pH; Figure S3: Photocurrent responses of (a) ZIF-67; (b) ZIF-67/MoS_2_; (c) ZIF-67@MoS_2_/rGO-modified GCEs in 0.1 mM and 0.2 mM glucose; (d) Current increment at the potential of 0.3 V (vs. Ag/AgCl) for ZIF-67, ZIF-67/MoS_2_ and ZIF-67@MoS_2_/rGO-modified GCEs in 0.1 mM and 0.2 mM glucose; Figure S4: The repeatability of the electrodes of ZIF-67@MoS_2_/rGO-modified GCEs; Figure S5: The selectivity of ZIF-67@MoS_2_/rGO-modified GCEs; Figure S6: The selectivity of ZIF-67@MoS_2_/rGO-modified SPEs; Table S1: The comparison of electrodes of ZIF-67@MoS_2_/rGO GCE and other photoelectrochemical glucose sensors; Table S2: Glucose detection in 5% human serum with ZIF-67@MoS_2_/rGO GCE; Table S3: The comparison of the ZIF-67@MoS_2_/rGO SPE and other photoelectrochemical glucose sensors.
