# Supplementary material for: ZIF-67 Anchored on MoS2/rGO Heterostructure for Non-Enzymatic and Visible-Light-Sensitive Photoelectrochemical Biosensing
Source: Biosensors (Basel). 2024 Jan 12;14(1):38. doi: 10.3390/bios14010038 (PMC10813494; doi:10.3390/bios14010038)
Supplement: Supplementary file 1 [file biosensors-14-00038-s001.zip › biosensors-2739571-supplementary.pdf]

Supporting Information

This file contains:

Supporting figures: Figure S1~S6.

Supporting tables: Table S1~S3.

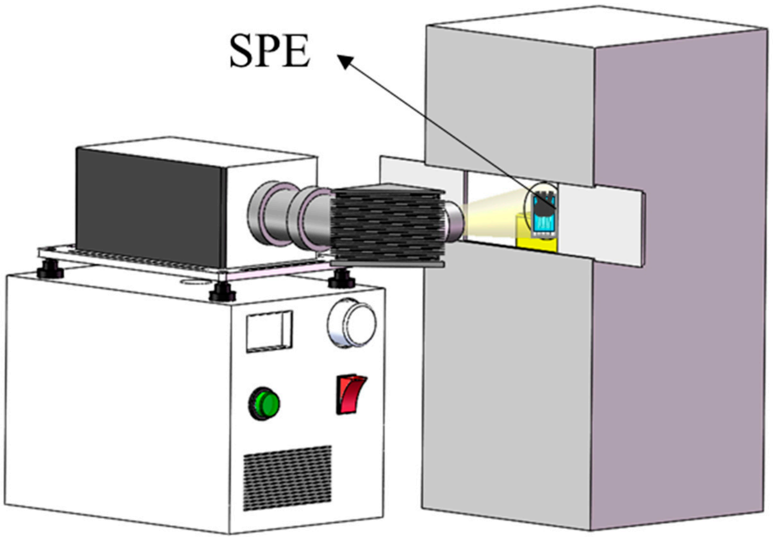

Figure S1. Schematic diagram of the photoelectrochemical laboratory bench

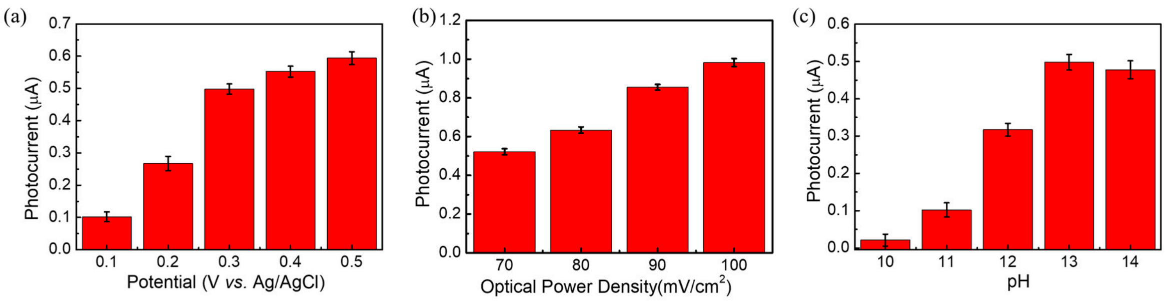

Figure S2. Optimization of (a) detection potential; (b) optical power density; (c) pH.

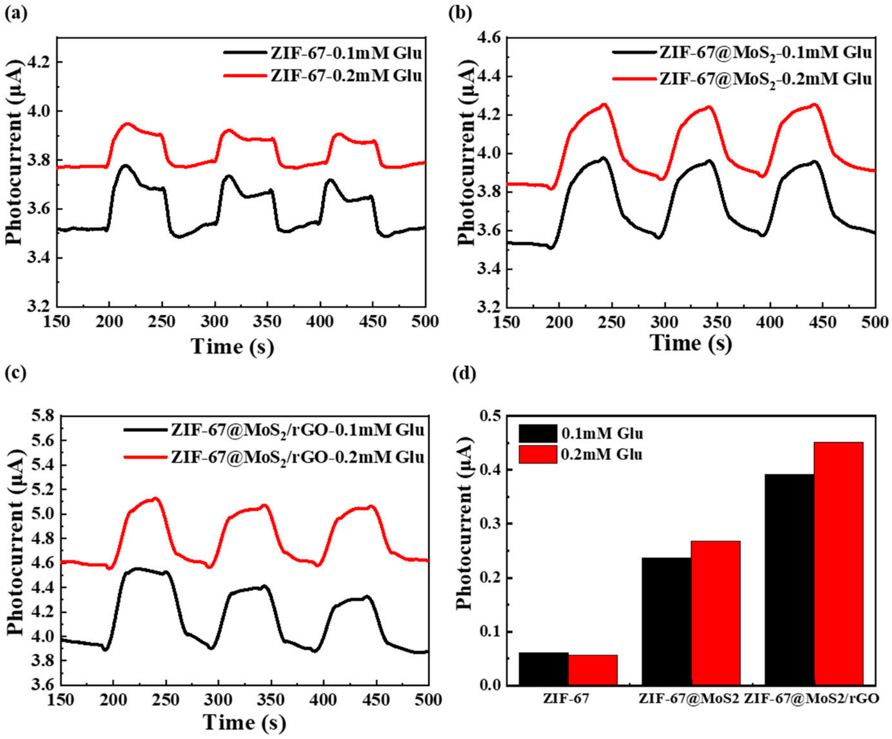

Figure S3. Photocurrent responses of (a) ZIF-67-; (b) ZIF-67/MoS_2-; (c) ZIF-67@MoS_2/rGO-modified GCEs in 0.1 mM and 0.2 mM glucose. (d) Photocurrent increment at the potential of 0.3 V (*vs.* Ag/AgCl) for ZIF-67, ZIF-67/MoS_2- and ZIF-67@MoS2/rGO-modified GCEs in 0.1 mM and 0.2 mM glucose

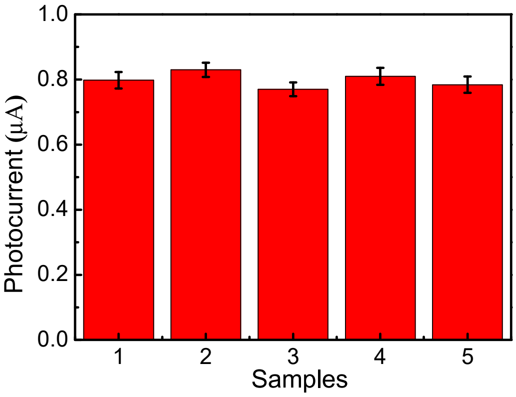

Figure S4. The repeatability of the electrodes of ZIF-67@MoS_2/rGO modified GCEs.

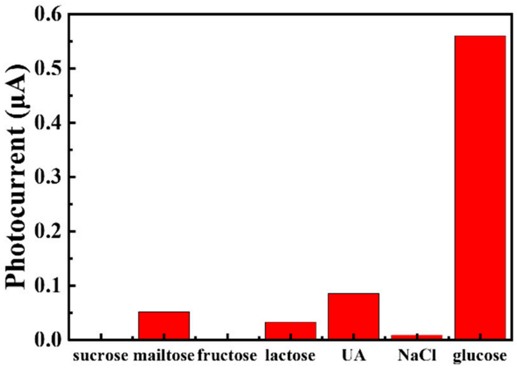

Figure S5. The selectivity of ZIF-67@MoS_2/rGO modified GCEs

The selectivity of ZIF-67@MoS_2/rGO GCEs for the photoelectrochemical detection of glucose was verified. The photocurrent response of the electrode was recorded by adding 0.5 mM sucrose, maltose, fructose, lactose, UA and NaCl in 0.1 M NaOH solution, and then 0.1 mM glucose was added to compare the response with other interfering substances.

As shown in **Figure S5**, it is obvious that there are only small photocurrent responses after the addition of interfering substances, which are almost negligible compared with the response of glucose. Considering that the concentration of interfering substances is 5 times that of glucose, the photocurrent response of glucose is still the most significant, indicating that the detection of glucose has a good selectivity, and can detect glucose in a reletively complex environment.

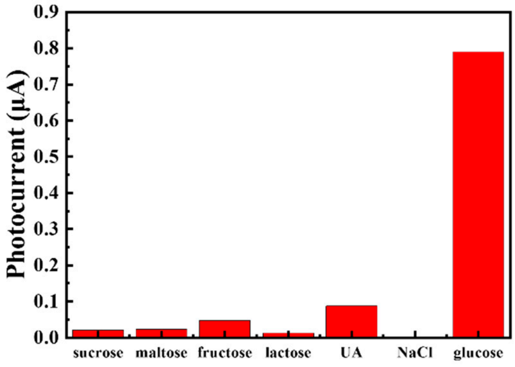

Figure S6. The selectivity of ZIF-67@MoS_2/rGO modified SPEs

The selectivity of ZIF-67@MoS_2/rGO SPEs for the photoelectrochemical detection of glucose was verified. The photocurrent response of the sensor was recorded by adding 0.5 mM sucrose, maltose, fructose, lactose, UA and NaCl into 0.1 M NaOH solution, respectively, and then 0.1 mM glucose was added to compare the response with other interfering substances. The results are shown in **Figure S6**.

It can be clearly seen that there are only small photocurrent responses after the addition of interfering substances, which are negligible compared with the response of glucose. Considering that the concentration of interfering substances is 5 times that of glucose, the photocurrent response of glucose is still the most significant, indicating that the detection of glucose has a good selectivity, and can detect glucose in a relatively complex environment.

Table S1. The comparison of electrodes of ZIF-67@MoS_2/rGO GCE and other PEC glucose electrodes

Modified electrode	Liner range (mM)	LOD (μM)	Refs
TiO_2/GO_x/Ti wire mesh	0-2.0	8.7	**[s1]**
ZnO/Au/Cu_2O	1-19	80	**[s2]**
Graphene-CdS	0.1-4.0	7	**[s3]**
PNR/ $ZnIn_2S_4$	0.08-30	27	**[s4]**
TiO_2 Nanotube Arrays	0.01-1.2	2.7	**[s5]**
Cu/ZnO Nano-Thorn	0.1-4.5	3.762	**[s6]**
ZIF-67@MoS_2/rGO GCE	0.05-4.5	3.9	This work

Table S2. Glucose detection in 5% human serum with ZIF-67@MoS_2/rGO GCE

Real Sample	Fitted Value (μA)	Scalar Addition (μA)	estimated value (μA)	Recovery (%)
1	0.0447	0.0892	0.132	97.9
2	0.0398	0.0892	0.134	105.6
3	0.0525	0.0892	0.141	99.2

The practical application of ZIF-67@MoS_2/rGO GCE was investigated by testing glucose concentrations in human serum samples which were provided by Zhongda Hospital in Nanjing, China. Before photoelectrochemical testing, the cryo-preserved sample was thawed at room temperature, and a certain amount was added into 0.1 M NaOH electrolyte with a pipetting gun to form 5% human serum sample. The spike and recovery method has been employed and analytical results are listed in **Table S2**.

Table S3. The comparison of the ZIF-67@MoS_2/rGO SPE glucose sensor and other PEC glucose sensors

Modified electrode	Liner range (mM)	LOD (μM)	Refs
GOD-SG/ZnS/ITO	0.2-5.5	40	**[s7]**
Fe_2O_3NR/FTO	0.5-2.5	5.5	**[s8]**
TiO_2 NRA/FTO	0.01-1.0	3.2	**[s9]**
A-TiO_2@GO_x FTO	1-20	19	**[s10]**
Gox/g-C_3N_4-TiO_2/ITO	0.05-16	10	**[s11]**
ZIF-67@MoS_2/rGO SPE	0.1-5.0	1.39	This work

[s1] Yang, W., Wang, X., Hao, W., Wu, Q., Peng, J., Tu, J., Cao, Y. 3D hollow-out TiO_2 nanowire cluster/GO_x as an ultrasensitive photoelectrochemical glucose biosensor. *J. Mater. Chem.B* **2020**, 8, 2363-2370.

[s2] Chen, D., Wang, X., Zhang, K., Cao, Y., Tu, J., Xiao, D., Wu, Q. Glucose photoelectrochemical enzyme sensor based on competitive reaction of ascorbic acid. *Biosens. Bioelectron.* **2020**, 166, 112466.

[s3] Zhang, X., Xu, F., Zhao, B., Ji, X., Yao, Y., Wu, D., Gao, Z., Jiang, K. Synthesis of CdS quantum dots decorated graphene nanosheets and non-enzymatic photoelectrochemical detection of glucose. *Electrochim. Acta* **2014**, 133, 615-622.

[s4] Yang, Y., Yang, J., He, Y., Li, Y. A dual-signal mode ratiometric photoelectrochemical sensor based on voltage-resolved strategy for glucose detection. *Sensor. Actuat. B-chem.* **2021**, 330, 129302.

[s5] Feng, C., Xu, G., Liu, H., Lv, J., Zheng, Z., Wu, Y. A flow-injection photoelectrochemical sensor based on TiO_2 nanotube arrays for organic compound detection. *J. Electrochem. Soc.* **2013**, 161, H57-H61.

[s6] Yang, B., Han, N., Hu, S., Zhang, L., Yi, S., Zhang, Z., Wang, Y., Chen, D., Gao, Y. Cu/ZnO nano-thorn with modifiable morphology for photoelectrochemical detection of glucose. *J. Electrochem. Soc.* **2021**, 168, 027516.

[s7] Du, J., Yu, X., Wu, Y., Di, J. ZnS nanoparticles electrodeposited onto ITO electrode as a platform for fabrication of enzyme-based biosensors of glucose. *Mater. Sci. Eng. C-Mater.* **2013**, 33, 2031-2036.

[s8] He, L., Zhang, Q., Gong, C., Liu, H., Hu, F., Zhong, F., Wang, G., Su, H., Wen, S., Xiang, S., Zhang, B. The dual-function of hematite-based photoelectrochemical sensor for solar-to-electricity conversion and self-powered glucose detection. *Sensor. Actuat. B-chem.* **2020**, 310, 127842.

[s9] He, L., Liu, Q., Zhang, S., Zhang, X., Gong, C., Shu, H., Wang, G., Liu, H., Wen, S., Zhang, B. High sensitivity of TiO_2 nanorod array electrode for photoelectrochemical glucose sensor and its photo fuel cell application. *Electrochem. Commun.* **2018**, 94, 18-22.

[s10] Yan, B., Zhuang, Y., Jiang, Y., Xu, W., Chen, Y., Tu, J., Wang, X., Wu, Q. Enhanced photoeletrochemical biosensing performance from rutile nanorod/anatase nanowire junction array. *Appl. Surf. Sci.* **2018**, 458, 382-388.

[s11] Liu, P., Huo, X., Tang, Y., Xu, J., Liu, X., Wong, D. K. A TiO_2 nanosheet-g-C_3N_4 composite photoelectrochemical enzyme biosensor excitable by visible irradiation. *Anal. Chim. Acta* **2017**, 984, 86-95.
